# Cervical Schwannoma: Diagnosis and Treatment in a Second-Level Hospital in Mexico

**DOI:** 10.7759/cureus.87481

**Published:** 2025-07-07

**Authors:** Ricardo Galaz Hernández, Alexis A Granados Flores, Jesus M Garcia Palazuelos Ramirez, Tania C Rodriguez Madrid, Jose R Gonzalez Soto, Dorian I Arriola Rios, Mayra C Sierra Reyes, Perla G Rubio Vega

**Affiliations:** 1 Surgery, Hospital General Regional No. 66, Juárez, MEX; 2 Surgery, Hospital General Regional No. 2, Juárez, MEX; 3 Surgery, Institute of State Workers Social Security and Social Services (ISSSTE), Juárez, MEX

**Keywords:** general surgery, head and neck tumors, mexico, schwannoma, surgical management

## Abstract

Cervical schwannoma is a rare, benign neurogenic tumor originating from Schwann cells, commonly located in the parapharyngeal space and associated with the vagus nerve. Its diagnosis often requires clinical examination, imaging, and histopathological confirmation. We report the case of a 22-year-old female presenting with a progressively enlarging, painless cervical mass. Imaging revealed a parapharyngeal lesion suggestive of a benign tumor. Surgical excision via cervicotomy was performed. Postoperatively, the patient developed transient dysphonia and intermittent dysphagia, managed with multidisciplinary support including speech therapy. Histopathology confirmed the diagnosis of schwannoma. Complete surgical resection is the treatment of choice for cervical schwannomas. However, their anatomical location poses a risk for nerve injury and postoperative complications. Early recognition, meticulous surgical technique, and comprehensive postoperative care are essential to minimize morbidity and ensure favorable outcomes.This case highlights the importance of including schwannoma in the differential diagnosis of cervical masses and underscores the value of coordinated surgical and rehabilitative management in achieving good functional recovery and prognosis.

## Introduction

Neurogenic tumors of the neck represent a rare group of benign neoplasms, with schwannoma being the most common lesion in this category, comprising about 25% to 45% of cases [1,2]. Schwannoma, also known as neurilemoma, is a benign tumor that originates from Schwann cells [3]. This tumor grows slowly and may affect any peripheral nerve, although it is most frequently found in the cervical region, particularly in the parapharyngeal space and in association with the vagus nerve [4,5]. A painless cervical mass that gradually grows larger is the typical clinical presentation and may cause compressive symptoms such as dysphagia, dysphonia or dyspnea [6,7]. In most cases, diagnosis is based on clinical examination and imaging studies, mainly computed tomography and magnetic resonance imaging, which allow characterization of the lesion including size, extent and relationship to surrounding structures [8,9].

Although the exact etiology of schwannoma remains unknown, it has been associated in some cases with mutations in the neurofibromatosis type 2 (NF2) gene, which encodes merlin, a tumor suppressor protein [10]. However, the majority of cases are isolated and have no family history [11].

The differential diagnosis includes branchial cleft cysts, pleomorphic adenomas, lymphadenopathies, and other benign or malignant neck tumors. Thus, clinical and radiological correlation, along with histopathological confirmation, are essential for appropriate management [12,13].

According to histopathology, schwannoma exhibits two growth patterns: Antoni A and Antoni B. Antoni A areas show densely cellular regions with elongated cells arranged in fascicles, often forming Verocay bodies. Antoni B areas consist of looser tissue, with a myxoid matrix and dispersed cells. Immunohistochemistry for S-100 protein is typically positive, reflecting Schwann cell origin and confirming the diagnosis [14].

Surgical intervention is the treatment of choice and should be carefully planned to preserve as much nerve function as possible. Meticulous dissection allows for complete tumor resection, although in some cases, functional sequelae related to damage to adjacent nerves may occur. The most common postoperative complications include dysphonia, dysphagia, and dyspnea, which may require multidisciplinary management by a team that may include a psychologist, speech therapist, rehabilitation physician, and otolaryngologist. Recurrence after complete resection is rare, and prognosis is generally favorable [15].

## Case presentation

A 22-year-old female patient began experiencing symptoms three months before the general surgery evaluation (July 2023), with a sensation of increasing volume in the right cervical region. The mass progressively enlarged until she reported mild pain (3/10 Numerical Pain Rating Scale (NPRS)), with no relieving factors and exacerbated by lateral neck movement, described as a pressing sensation. She denied associated symptoms such as dysphagia, dyspnea, or fever. Her personal history was negative for smoking, alcohol consumption, or illicit drug use. She also had no history of chronic degenerative diseases, surgeries, allergies, or blood transfusions.

On physical examination, a deep-seated mass with a firm consistency was found in the carotid triangle of the neck, measuring approximately 4 × 3 × 3 cm. It was non-tender, without increased temperature, no signs of fluid discharge, and no changes in the overlying skin coloration

At this time, the patient denied dyspnea and showed no signs of constitutional syndrome or systemic inflammatory response. This case demonstrates a typical presentation of cervical schwannoma with delayed onset of mild compressive symptoms.

**Figure 1:**
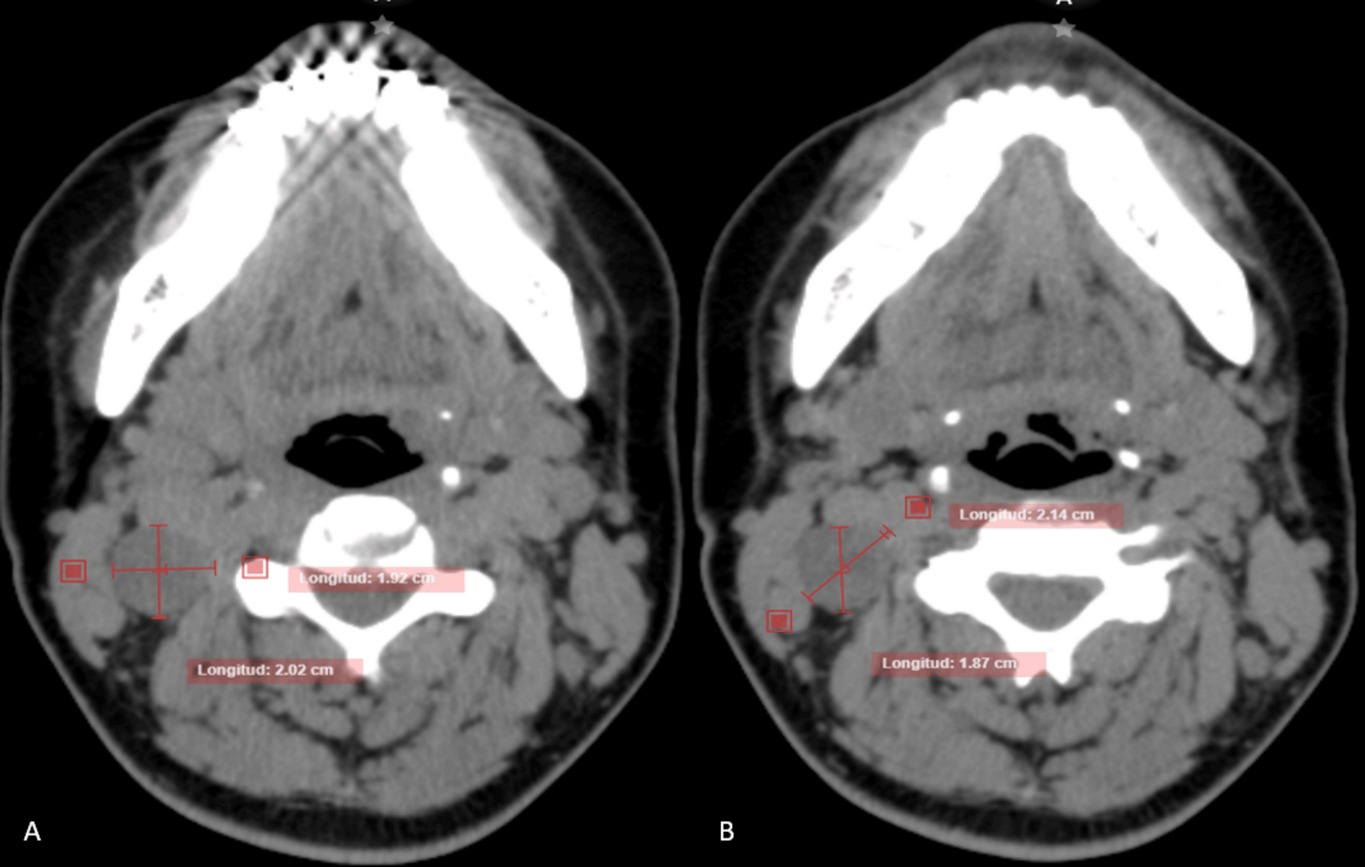
Axial CT slices of the head and neck shows a homogeneous, ovoid-shaped parapharyngeal mass. A) Measures 1.92x2.02 cm B) Measures 2.14x1.87 cm

As part of the diagnostic workup, a Computed Tomography (CT) scan of the head, neck, and thorax was ordered. Findings included a rounded, solid-appearing, heterogeneous parapharyngeal mass in the right region, measuring 2.02 × 1.92 × 2.67 cm, with no central calcifications or irregular densities (HU range 44-48). No lymphadenopathy or pathology was found on the contralateral side. Based on these findings, a differential diagnosis of parapharyngeal cyst, branchial cleft cyst, or pleomorphic adenoma was considered (Figure 1). A routine preoperative protocol, including laboratory tests, chest X-ray, and electrocardiogram, was completed, and the patient was scheduled for surgery.

Surgical procedure and postoperative course

**Figure 2:**
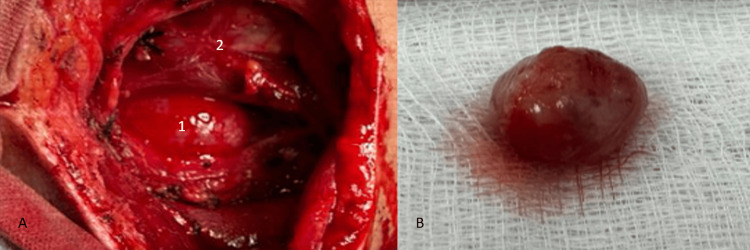
A) Parapharyngeal tumor located posterior to the carotid vascular bundle, B) Tumor specimen with smooth borders, firm consistency, and yellow-whitish coloration. 1) Tumor 2) Carotid artery and jugular vein

On the day of surgery, the patient underwent an anesthetic evaluation and was subsequently taken to the operating room. Under Rossier's position and following standard aseptic and antiseptic protocols for the surgical field, a Paul André-type cervicotomy incision (hockey stick) was made. Dissection was carried out through the skin and subcutaneous tissue, followed by the creation of a subplatysmal flap, which continued in the anterior region of the sternocleidomastoid muscle, within the carotid triangle, where the carotid artery and internal jugular vein were identified. Immediately posterior to these structures, a tumor measuring approximately 2.5 × 2 × 2 cm was found (Figure 2A), attached to the vagus nerve (cranial nerve X).

Meticulous dissection of the tumor was performed to preserve the nerve as much as possible. The lesion was successfully excised and sent to pathology for definitive diagnosis and assessment of the tumor's nature (Figure 2B). Hemostasis was achieved, and closure was performed in anatomical layers: fascia with 2-0 Vicryl, and skin with 3-0 nylon using intradermal sutures.

Postoperatively, the patient was transferred to the general surgery ward and continued with satisfactory progress; no respiratory symptoms or swallowing difficulties were shown. The surgical site showed no signs of bleeding, hematoma, or dehiscence, with minimal pain during lateral neck movement. Twenty-four hours later she was discharged.

Follow-up was conducted through the general surgery outpatient clinic at 15 days, one month, three months, and subsequently every six months. At the first follow-up visit, the patient reported postoperative dysphonia, intermittent dysphagia, and dyspnea on moderate exertion. Consequently, referrals were made to otolaryngology, neurosurgery, and phoniatrics services, and she was enrolled in speech therapy. She is currently showing improvement in symptoms.

**Figure 3:**
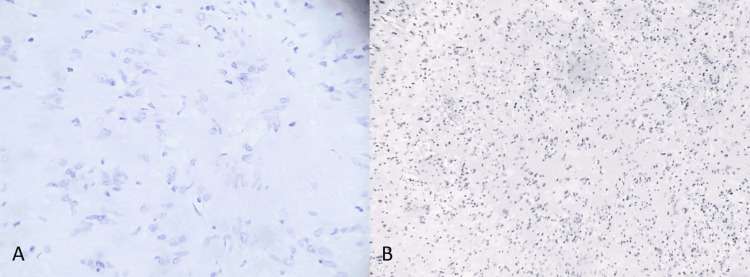
A) Microscopic view under high dry objective with hematoxylin stain, showing palisading nuclei and poorly differentiated fibrillar cytoplasm, B) Histological section showing hypercellular areas with ovoid nuclei and Verocay bodies (Antoni A areas), as well as hypocellular, diffusely loose Antoni B areas.

Histopathological analysis reported a cervical mass with microscopic characteristics compatible with schwannoma (Figure 3A, 3B).

## Discussion

Cervical schwannoma is a benign tumor derived from Schwann cells which, although rare, represents an important diagnostic consideration in cervical masses, particularly in young adults without significant clinical history, as exemplified by our 22-year-old patient [1,2]. The typical clinical presentation of schwannoma is a painless, slowly and progressively growing cervical mass without inflammatory or systemic signs, as was observed in this case. This may lead to delayed diagnosis due to the insidious nature and nonspecific initial symptoms [3,4].

The identification of a mass located within the carotid triangle and its direct relationship with the vagus nerve poses a significant surgical challenge. This anatomical region is complex due to the concentration of vital neurovascular structures, including the carotid artery, internal jugular vein, and vagus nerve, which must be preserved to avoid serious complications. In this context, the choice of surgical technique - in this case, a Paul André-type cervicotomy with meticulous dissection and maximal preservation of the vagus nerve - was crucial for the success of the procedure. This incision was preferred over other approaches, such as the Lahey, Conley, or Attie incisions, because it offers superior exposure due to the tumor’s location [5,6].

The complete resection of the schwannoma is the standard management. However, surgical manipulation frequently results in an injury that may cause temporary or permanent functional losses because of the tumor's proximity to the nerve. Postoperative complications such as dysphonia, intermittent dysphagia, and exertional dyspnea are well-documented in the literature. Early referral to speech therapy and multidisciplinary care facilitated progressive symptom improvement, showing the importance of follow-up after surgery [7,13].

Histopathologically, schwannomas are characterized by Antoni A and Antoni B cellular patterns and strong S-100 protein positivity, confirming their Schwann cell origin and helping differentiate them from other cervical neoplasms. Diagnostic confirmation via biopsy or postoperative pathological study is essential for appropriate treatment and favorable prognosis [14].

In an effort to maximize clinical and functional results, this case emphasizes the significance of a multifaceted approach that includes precise surgical planning, accurate imaging diagnosis, and postoperative follow-up. Additionally, it highlights that although schwannomas are benign, their anatomical location can make resection challenging and lead to potential side effects that require specialized care. The clinical experience with this patient reinforces that surgery remains the cornerstone of treatment for cervical schwannomas, always accompanied by careful assessment of risks and benefits and a postsurgical rehabilitation strategy to ensure proper functional recovery. Accurate tumor identification, appropriate surgical technique, and complication management lead to an excellent prognosis, with low recurrence probability and good quality of life for the patient [15].

## Conclusions

Schwannoma is an uncommon cervical tumor; however, its location involves anatomically significant considerations due to the numerous visceral, vascular, and neural structures found in the neck. The evaluation of a patient presenting with a cervical mass must be conducted cautiously, including detailed physical examination and appropriate imaging studies. Histopathological diagnosis is essential for guiding medical management, as well as for determining the prognosis and appropriate follow-up. It is crucial that general surgeons, head and neck surgeons, and surgical oncologists keep this differential diagnosis in mind, along with all the implications previously discussed in the diagnostic approach to a patient with a neck tumor. Prompt recognition and expert surgical management remain the cornerstones for favorable outcomes in patients with cervical schwannomas.

## References

[1] Farma JM, Porpiglia AS, Vo ET (2022). Benign neurogenic tumors. Surg Clin North Am.

[2] Albert P, Patel J, Badawy K, Weissinger W, Brenner M, Bourhill I, Parnell J (2017). Peripheral nerve schwannoma: a review of varying clinical presentations and imaging findings. J Foot Ankle Surg.

[3] Herrera Valenzuela JA, García Palazuelos JM, Nava Coronado A, Lujan Terrazas LB (2022). Schwannoma cervical. Cirujano General.

[4] Knight DM, Birch R, Pringle J (2007). Benign solitary schwannomas: a review of 234 cases. J Bone Joint Surg Br.

[5] Katre MI, Telang RA (2015). Schwannoma of parapharyngeal space: a case report. Indian J Surg.

[6] Nao EE, Dassonville O, Bozec A (2012). Cervical sympathetic chain schwannoma. Eur Ann Otorhinolaryngol Head Neck Dis.

[7] Yasumatsu R, Nakashima T, Miyazaki R, Segawa Y, Komune S (2013). Diagnosis and management of extracranial head and neck schwannomas: a review of 27 cases. Int J Otolaryngol.

[8] Pilavaki M, Chourmouzi D, Kiziridou A, Skordalaki A, Zarampoukas T, Drevelengas A (2004). Imaging of peripheral nerve sheath tumors with pathologic correlation: pictorial review. Eur J Radiol.

[9] Anil G, Tan TY (2010). Imaging characteristics of schwannoma of the cervical sympathetic chain: a review of 12 cases. AJNR Am J Neuroradiol.

[10] Vlashi R, Sun F, Zheng C, Zhang X, Liu J, Chen G (2024). The molecular biology of NF2/Merlin on tumorigenesis and development. FASEB J.

[11] Tamura R (2021). Current understanding of neurofibromatosis type 1, 2, and schwannomatosis. Int J Mol Sci.

[12] Li C, Wang DR, Sun RH, Zhang ZY (2025). [Current status and future directions of the diagnosis and treatment for head and neck tumor in China]. Zhonghua Yi Xue Za Zhi.

[13] Chen H, Guo Y, Li C, Zhou L (2022). Paraglottic space schwannoma: a case report and literature review. J Int Med Res.

[14] Belakhoua SM, Rodriguez FJ (2021). Diagnostic pathology of tumors of peripheral nerve. Neurosurgery.

[15] Ansari I, Ansari A, Graison AA, Patil AJ, Joshi H (2018). Head and neck schwannomas: a surgical challenge-a series of 5 case. Case Rep Otolaryngol.

